# A First-Class Simulation: In-Situ In-Flight Medical Emergencies Curriculum for Emergency Medicine Residents Aboard a Commercial Airliner

**DOI:** 10.7759/cureus.37562

**Published:** 2023-04-14

**Authors:** Madison B Kommor, Krystin N Miller, Thomas L Powell, Andrew M King, Christopher E San Miguel, Alix Delamare Fauvel, Jennifer A Frey, Jennifer Yee

**Affiliations:** 1 Department of Emergency Medicine, The Ohio State University Wexner Medical Center, Columbus, USA

**Keywords:** critical care, emergency medicine, in-flight emergencies, austere medicine, medical simulation

## Abstract

Introduction: In-flight medical emergencies occur in an estimated one out of 604 flights. Responding in this environment poses a unique set of challenges unfamiliar to most emergency medicine (EM) providers, including physical space and resource limitations. We developed a novel high-fidelity in-situ training curriculum focused on frequent or high-risk in-flight medical scenarios while replicating this austere environment.

Methods: Our residency program coordinated with our local airport’s chief of security and an airline-specific station manager to arrange the use of a grounded Boeing 737 commercial airliner during late evening/early morning hours. Eight stations reviewing in-flight medical emergency topics were reviewed, five of which were simulation scenarios. We created medical and first-aid kits that reflect equipment used by commercial airlines. Residents’ self-assessed competency and medical knowledge were assessed both initially and post-curriculum using a standardized questionnaire.

Results: Forty residents attended the educational event as learners. Self-assessed competency and medical knowledge increased after curriculum participation. All tested aspects of self-assessed competency had a statistically significant increase from a mean of 15.04 to 29.20 out of a maximum score of forty. The mean medical knowledge score increased from 4.65 to 6.93 out of 10 maximum points.

Conclusion: A five-hour in-situ curriculum for reviewing in-flight medical emergencies increased self-assessed competency and medical knowledge for EM and EM-internal medicine residents. The curriculum was overwhelmingly well-received by learners.

## Introduction

In-flight medical emergencies occur during approximately one of 604 flights with an average of 44,000 in-flight medical events worldwide annually [1]. The Aviation Medical Assistance Act of 1998 compels the Federal Aviation Administration (FAA) to maintain a list of medical supplies required to be carried aboard every commercial airliner within the United States. Each plane is required to have one medical kit and one to four first aid kits, depending on the number of passengers [2].

Flight attendants are trained in Basic Life Support resuscitative care, but many medical events will exceed the training of on-board airline staff, requiring assistance from passengers who have more advanced medical knowledge [3]. Physicians from other specialties have demonstrated varying levels of confidence [4,5] and willingness [4-6], caring for airline passengers requiring medical assessment and treatment. Emergency medicine (EM) physicians have the requisite knowledge and skillset to manage medical emergencies, however, doing so in a cramped environment, with limited medical supplies, and a lay audience is not routinely a part of EM training. This curriculum strives to replicate these resource limitations.

Previous publications of in-flight medical emergencies described individual simulation scenarios [7,8], a curriculum for medical students consisting of a lecture and a simulation scenario [9], a simulation-based curriculum for medical students participating in a wilderness and extreme environmental medicine elective [10], and use of a mobile phone application for assistance with management of in-flight medical emergency scenarios [11]. These educational interventions were held in standard simulation settings. To our knowledge, this is the first publication describing an educational curriculum for in-flight emergencies held on a commercial airliner.

To train EM physicians to assess and treat passengers in-flight, we developed a high-fidelity training curriculum focused on high-probability or high-acuity in-flight medical scenarios conducted aboard an actual airliner. The purpose was to execute an in-situ in-flight medical emergencies curriculum that reinforces resuscitative and management principles for managing common emergencies in this austere environment.

## Materials and methods

Study setting, design, and population

This study demonstrates the implementation of an in-flight medical emergency curriculum. The curriculum was designed for EM and emergency medicine-internal medicine (EM-IM) residents and employed in April 2022. The study was approved by the Ohio State University Wexner Medical Center - approval 2022B0164.

The study setting was a local international airport in conjunction with a United States-based global airline which included access to a passenger terminal and a Boeing 737 airliner. The training occurred while the airliner was on the ground, absent of passengers and flight crew. Participants included EM (three-year program) and EM-IM (five-year program) residents from a university-based, tertiary care teaching hospital. Each of the eight learning stations was facilitated by a paired faculty member and resident. Three simulation staff attended the event to run two 3G® SimMan manikins (Laerdal Medical, Wappingers Falls, NY). In total, 73 residents, fellows, faculty, and simulation staff attended.

Curriculum development

Our first step was to secure the use of a commercial airliner and establish airline and airport contacts. Local emergency management agencies who conduct regional airport system disaster exercises connected our residency education team with the airline-specific station manager (SM). The SM was our primary contact for obtaining airliner access and determining event feasibility. When approaching the SM, we outlined our proposed curriculum while highlighting public safety and public relations benefits for the airline and the community at large. 

Planning discussions with the SM and airport chief of security (COS) began six months before the event. The date was chosen by considering moderate temperatures with a low risk of inclement seasonal weather. Training was held overnight to minimize the impact on airline and airport resources. The last flight for the airline came in around 20:30. Our event ran from 22:00-03:00. The COS allowed us to navigate security parameters and coordinate with overnight airport security and parking services. Approval of residency and clinical leadership ensured that residents had protected time off from clinical duties.

Our faculty and simulation staff created medical and first-aid kits based on commercial airline regulations as outlined by United States Federal Law (121 C.F.R. § 121.803, 2005) (Table 1) [12]. An EM pharmacist provided empty medication vials for kits. 

**Table 1:** Medical kit contents Note: Does not reflect kit contents of any specific commercial airline. *Item not required by code of federal regulation IV=intravenous, g=gauge, cc=cubic centimeter, mL=milliliter, mg=milligram, pk=pack, PFS=preservative-free solution, ODT=oral dissolvable tablet, CPR=cardiopulmonary resuscitation, BVM=bag-valve mask, BP=blood pressure, fr=French, gm=gram

Equipment	# Per Kit		Total #
YELLOW BAG: IV EQUIPMENT			4
IV Start Kit			
Curaplex® IV start kit w/ tourniquet, non-latex*	1		4
Safety catheter, IV: 18g	1		4
Safety catheter, IV: 20g	1		4
Safety catheter, IV: 22g	2		8
Gloves, non-latex	2		8
Safety infusion butterfly, 21g*	2		8
Alcohol pads	4		16
Scissors, trauma* (tape scissors required by code of federal regulations)	1		4
Tape, 1” roll	1		4
Drape, surgical 18 inch x 26 inch*	1		4
IV administration tubing	1		4
Needle & Syringe Kit			
Safety needle 18g	1		4
Safety needle, 20g	2		8
Safety needle, 22g	6		24
Syringe, 1cc	2		8
Syringe, 5cc	2		8
Syringe, 10cc	1		4
Others			
Sodium chloride intravenous fluid, 0.9% 500 ml	1		4
Gloves, non-latex	2		8
Alcohol pads	4		16
ORANGE BAG: MEDICATIONS			4
Hypoglycemia Kit			
Dextrose injection 50%, 50 ml vial (IV use)	1		4
Syringe, 30cc	1		4
Safety needle, 18g	1		4
Pads, alcohol prep	2		8
Allergy Kit			
Diphenhydramine injection, 50mg/ml 1ml	2		8
Diphenhydramine, 25mg pills	4		16
Syringe, 1cc	2		8
Safety needle, 21g	2		8
Alcohol pads	2		8
Anaphylaxis Kit			
Epinephrine injection, 1:1,000 1ml	2		8
Syringes, 1cc	2		8
Safety needles, 21g	2	8	
Alcohol pads	2	8	
Atropine Cardiac Kit			
Atropine pre-filled syringe, 0.1 mg/ml 10ml (IV use)	2	8	
Alcohol pads	2	8	
Epinephrine Cardiac Kit			
Epinephrine pre-filled syringe 1:10,000 10ml	2	8	
Alcohol pads	2	8	
Miscellaneous			
Acetaminophen, 325 mg tablets (2/pk)	2	8	
Aspirin, 325 mg tablets (2/pk)	2	8	
Lidocaine single dose vial 2% 5ml or Lidocaine PFS 2% 20 mg/ml 5ml	2	8	
Bottle, nitroglycerin 0.4mg tablets (25/bottle)	1	4	
Ondansetron, 4mg ODT tablets*	6	24	
Naloxone 4mg nasal spray*	1	4	
Albuterol metered dose inhaler	1	4	
BLUE BAG: AIRWAY		4	
Airways, oropharyngeal (6 sizes)	6	24	
CPR/BVM masks (pediatric, small adult, large adult)	3	12	
Resuscitator (BVM), Adult	1	4	
Valve, one-way CPR	1	4	
RED BAG: FIRST AID KIT		1	
Adhesive bandage compresses, 1-inch	16	16	
Antiseptic swabs	20	20	
Ammonia inhalants	10	10	
Bandage compresses, 4-inch	8	8	
Triangular bandage compresses, 40-inch	5	5	
Arm splint, non-inflatable	1	1	
Leg splint, non-inflatable	1	1	
Roller bandage 4-inch	4	4	
Adhesive tape, 1-inch standard roll	2	2	
Bandage scissors	1	1	
BLACK BAG: DIAGNOSTIC EQUIPMENT		4	
Stethoscope	1	4	
BP Cuff	1	4	
PERIMETER (OUTSIDE OF BAGS)		4	
IV seals, green*	3	12	
Sharps container*	1	4	
Speedicath intermittent urinary catheter 14fr*	1	4	
Lubricant, gel 5gm*	2	8	
Usage form*	1	4	
Basic instructions for use of the drugs in the kit	1	4	

Learning station topics were chosen based on the possible need for flight diversion, frequency of reported in-flight occurrence [1,10], or if they were high-acuity pathologies that may be treated with equipment or medications commonly available on commercial in-flight medical kits. Five simulation-based learning stations included anaphylaxis, shockable rhythm arrest, syncope secondary to pulmonary embolism, anterior epistaxis, and an agitated passenger. Three discussion-based learning stations included aeromedical considerations, medicolegal topics, and what to do if oxygen masks drop/decompression illness.

Our EM faculty developed an eight-item self-assessed competency survey and a 10-item multiple-choice medical knowledge test that were given to the residents before and after the curriculum (Appendices). The competency survey was based on a five-point Likert scale. Questions for both assessment tools were identical both pre- and post-curriculum other than open-ended questions eliciting things that the residents enjoyed about the session and opportunities for improvement at the bottom of the post-curriculum competency survey. Pre-existing validated medical knowledge assessments for in-flight medical emergencies are not currently available in published literature.

Implementation phase

All participants underwent Transport Security Administration clearance. A list of materials was supplied to the SM and COS so items going into the airport could be monitored (Table 2).

**Table 2:** Equipment list AED=automated external defibrillator, FAA=Federal Aviation Administration

Equipment	Quantity	Description
SimMan® 3G Manikin	2	Full body manikin for use in simulation training. Technology is complex and involves lots of wiring and computer
Gurney & manikin storage bags	2 gurneys 2 storage bags	Standard ambulance gurney and storage bags for the 3G manikins
Laptop, wireless mouse	2 laptops 2 wireless mice	For control of 3G manikins during simulation. Standard laptop and mouse
Extension cables, power supply cables	2 extension cables 2 power supply cables	Standard extension cables and power supply cables.
Moulage	Various make-up items	Makeup to mimic injuries on manikins and individuals portraying patients.
Sheets	4	Standard sheets to cover the manikins
Bed Pads	2	Standard issue bed pads for liquid absorbent
AEDs	3	Simulated AEDs & associated cardboard vitals
Medication Kit	4	Demo versions of Airlines Emergency Medical Kit
First Aid Kit	1	Demo version of FAA First Aid kit
Zipties	1 bag	Demonstration of securing an agitated passenger safely
EpiPen® task trainers	2	Practice administering EpiPens®
Trauma shears	1 set	To cut off any applied zipties
Tampons	2	To demonstrate epistaxis treatment

The simulation staff arrived early for setup with a simulation vehicle displaying a university affiliation magnet. These staff were escorted onto the tarmac. Participants were escorted through security by the SM and COS. Once at the gate, the SM addressed safety precautions, including where to meet and how to exit the plane in case of an emergency. Entry into the flight deck was prohibited. During this time, facilitators set up their respective learning stations. Residents were assigned into groups, which were created to accommodate equal mixes of post-graduate level of training.

Learning station topics, locations, and topics are described in Table 3. Residents had approximately twenty minutes at each station, including debriefing led by the station’s facilitating resident and faculty member. Debriefing sessions focused on exploring observed behaviors of each team and reviewing optimal management strategies. Four out of the five simulation scenarios occurred on the airliner (Figure 1). Residents rotated from front to back of the plane. Once residents completed the agitated patient station, they deplaned using rear airliner stairs, were escorted by security back to the jet bridge, and then continued with the gate-based stations.

**Table 3:** Station descriptions

#	Station Topic	Location	Topics Covered	Equipment
1	Shockable Rhythm Arrest	Front galley	Diversion considerations, Medication options, Performing chest compressions in enclosed spaces	Medication kit, 3G manikin, AED
2	Syncope/Pulmonary Embolism	Forward passenger cabin	Most common in-flight emergency, Differential diagnosis, Equipment readily available for work-up	Apple watch, Medication kit, 3G manikin with bruising moulage to forehead, AED
3	Epistaxis	Middle passenger cabin	Alternative treatments when standard packing isn’t available, Administration of local anesthetics (eg: atomized lidocaine through IV tubing)	Medication kit, First aid kit, Blood moulage for confederate patient, Tampons
4	Agitated Passenger	Rear passenger cabin	Verbal de-escalation, Assessing for scene safety, Medications available for sedation	Zipties, Trauma shears
5	Medicolegal Aspects	Gate area in terminal	Domestic versus international flights and provider protection, Offered compensation, Documentation of in-flight events, Alcohol ingestion and rendering care	
6	Aeromedical Considerations	Gate area in terminal	Diversion considerations, Physical space needed to render care, Air-filled balloons decreasing with descent (endotracheal tubes for medical transport), Communication with flight crew/ground control	
7	Dropped Oxygen Masks	Gate area in terminal	Cabin pressurization, Cabin altitude which masks were dropped, Decompression illness symptoms	Oxygen mask (airline provided)
8	Anaphylaxis	Gate area in terminal	Medications available, Crisis resource management/asking passengers for medications	Medication kit, EpiPen simulators

**Figure 1:**
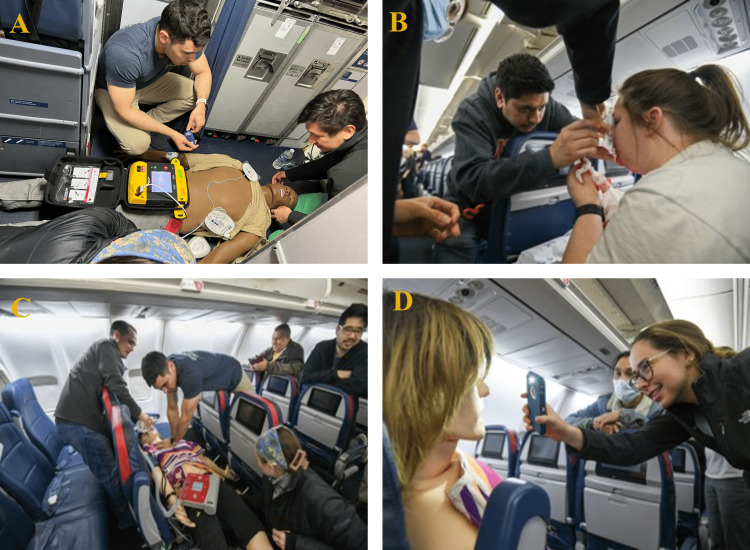
On-board simulation scenarios Top left (A): Shockable rhythm arrest station in the front cabin. Top right (B): Epistaxis station in mid-passenger cabin. Bottom left (C) and right (D): Syncope/pulmonary embolism station in the mid-passenger cabin.

## Results

Forty-eight total residents attended the curriculum, forty (83.3%) as learners including thirty-three EM residents and seven EM-IM residents. Eight residents served as learning station co-facilitators. Resident learners included fifteen (37.5%) women and twenty-five (62.5%) men. Three fellows, one EM clinical pharmacist, and nine faculty attended as learners.

Medical knowledge scores increased from a mean total score of 4.65 out of ten points for pre-testing to a mean total score of 6.93 for post-testing (Table 4). The largest score increase was for advanced cardiovascular life support (ACLS) medication availability in-flight and the most common type of in-flight emergencies, followed by questions regarding oxygen mask deployment and medicolegal considerations for rendering in-flight care. Self-assessed competency increased from a mean pre-curriculum score of 15.04 (range 8-23 out of a maximum score of forty) to a mean post-curriculum score of 29.20 (range 21-36 out of a maximum score of forty) with all assessed items reaching statistical significance (Table 5).

**Table 4:** Medical knowledge assessment results MK=medical knowledge

Question	Pre-test success n (%) N=40	Post-test success n (%) N=40	p-value
MK1	35 (87.5)	39 (97.5)	0.046
MK2	23 (57.5)	26 (65.0)	0.317
MK3	10 (25.0)	15 (37.5)	0.132
MK4	5 (12.5)	24 (60.0)	<0.001
MK5	24 (60.0)	38 (95.0)	<0.001
MK6	7 (18.0)	20 (50.0)	0.001
MK7	39 (97.5)	39 (97.5)	0.317
MK8	15 (38.5)	30 (75.0)	0.001
MK9	19 (48.7)	30 (75.0)	0.008
MK10	10 (25.6)	17 (42.5)	0.083

**Table 5:** Self-assessed competency results SD=standard deviation

Items	Pre-test Mean (SD)	Post-test Mean (SD)	p-value
Describing what medications may be found in an in-flight medical care bag.	1.59 (0.64)	3.70 (0.57)	<0.001
Describing what equipment may be found in an in-flight medical care bag	1.54 (0.65)	3.70 (0.57)	<0.001
Performing an initial assessment on a patient in-flight	2.86 (1.02)	4.00 (0.53)	<0.001
Obtaining vitals on a patient in-flight	2.96 (0.97)	4.03 (0.64)	<0.001
Making recommendations regarding disposition/potential need for diversion when discussing the case with the pilot/ground control	2.08 (0.89)	3.59 (0.50)	<0.001
Describing appropriate laws for rendering care and physician liability in the United States for in-flight emergencies	1.24 (0.55)	3.51 (0.65)	<0.001
Describing appropriate laws for rendering care and physician liability internationally for in-flight emergencies	1.24 (0.49)	3.19 (0.81)	<0.001
Describing considerations for care that may change for patients being transferred by air ambulance (eg. Medflight)	1.59 (0.64)	3.49 (0.56)	<0.001

## Discussion

Although not legally obligated to render aid in the United States, the commercial airline industry relies on healthcare professional passengers to render care for acutely ill co-passengers. In order to prepare our EM and EM-IM residents to treat in-flight emergencies, we developed a novel in-situ simulation-based curriculum. Resident self-assessed competencies were highest for obtaining vitals on flight both pre- and post-curriculum and were lowest for describing legal aspects of rendering care and physician liability. However, all assessed topics for self-assessed competency had statistically significant increases from pre- to post-curriculum. The largest gains in medical knowledge were related to medications available on a flight and on relevant flight operations.

To date, the described curriculum is the only model successfully performed aboard an actual commercial airliner. Conducting the training on an aircraft allowed for learners to appreciate rendering care in a cramped environment with ergonomic considerations not typically encountered in the emergency department and difficult to replicate in a typical simulation laboratory. The benefits of in-situ simulation, including human factors impact of practicing in the expected clinical space, have been supported by multiple studies [13,14]. Operating in this unique training environment offered participants a chance to practice in-flight resuscitation with a level of immersion and ergonomic accuracy not yet previously described. Overwhelmingly positive qualitative feedback obtained on the post-curriculum assessment survey highlighted the perceived value of assessing passengers in the actual physical space encountered on a commercial flight.

Previously published in-flight medical emergency simulation scenarios were held in a dedicated simulation center or educational facilities [7-11]. One utilized a critical actions checklist for performance assessment but did not report these results [7]. A hypoglycemia in-flight emergency simulation case did not report any objective or competency-based results from their training [8]. One group assessed the perceived confidence and comfort of fourth-year medical students [9], and two utilized unvalidated medical knowledge exams [9,10]. Lastly, one study evaluated two scenarios using scenario-specific checklists, measured time to critical actions, global rating scores, and self-assessment readiness and willingness surveys [11]. Checklists were internally cross-checked for content validity by the authors, but they noted that external validity checks could not be performed due to a lack of existing performance expectations for those responding to in-flight emergencies.

Our curriculum implementation was facilitated by our first author and other EM faculty who were familiar with local emergency management agencies. Interested parties may reach out to those who conduct regional disaster drills for these contacts. Once airline-specific SM and airport COS personnel are identified, reviewing mutually beneficial outcomes such as positive publicity for the airline and airport, increasing in-flight medical emergency management skills for passengers, and strengthening connections which may be used for regional disaster drills may be useful.

EM physicians are well suited to assist during in-flight medical emergency scenarios. All medical providers called upon to provide care on an airliner should be resourceful and utilize medical kits and supplies such as ice, other passengers’ medications, and the in-flight communication system to receive assistance from on-call ground medical providers.

Limitations

The medical knowledge test and self-assessed competency survey were unvalidated, included a short test/retest interval, and utilized lower-level Kirkpatrick assessments [15].

The number of scenarios was limited by physical space, leading to six to seven learners per group. Group sizes may have decreased the amount of hands-on time at each station. Two of our simulation scenarios included cardiopulmonary resuscitation, which led to the inclusion of two 3G® SimMan manikins, ambulance gurneys for transport, laptops and wireless mice for manikin control, and extension cables with supply cables for power. It would be more resource-effective to use confederates.

Curriculum execution may be challenging for programs near airports with high flight traffic. We worked with one airline, so our experience may not extrapolate similarly with other airlines. Undoubtedly, different airports have varying security protocols to be navigated. In addition, the successful execution of simulations like this hinges on some factors that are simply out of the control of those planning the event. Weather conditions delaying incoming flights, after-hours airport staffing, and miscellaneous security concerns have the potential to interfere with successful simulation execution. 

Future directions

One suggestion was to use radios to contact confederate pilots or medical ground control to initiate the discussion regarding diversion. Other cases may involve management of pediatric passengers, treating multiple simultaneous patients after a turbulence event, or involvement of emergency medical services. Medical students and interdisciplinary teams may also benefit from this training framework.

## Conclusions

A five-hour in-situ in-flight medical emergencies curriculum on a commercial airliner was well received by residents and improved knowledge and self-reported competency. Learners most appreciated the in-situ setting and simulation-based scenarios. Establishing early contact with the SM and COS was crucial for event planning. Hosting the event at a low airline traffic period allowed for ease of movement within the airport and through security. 

## Figures and Tables

**Figure 2:**
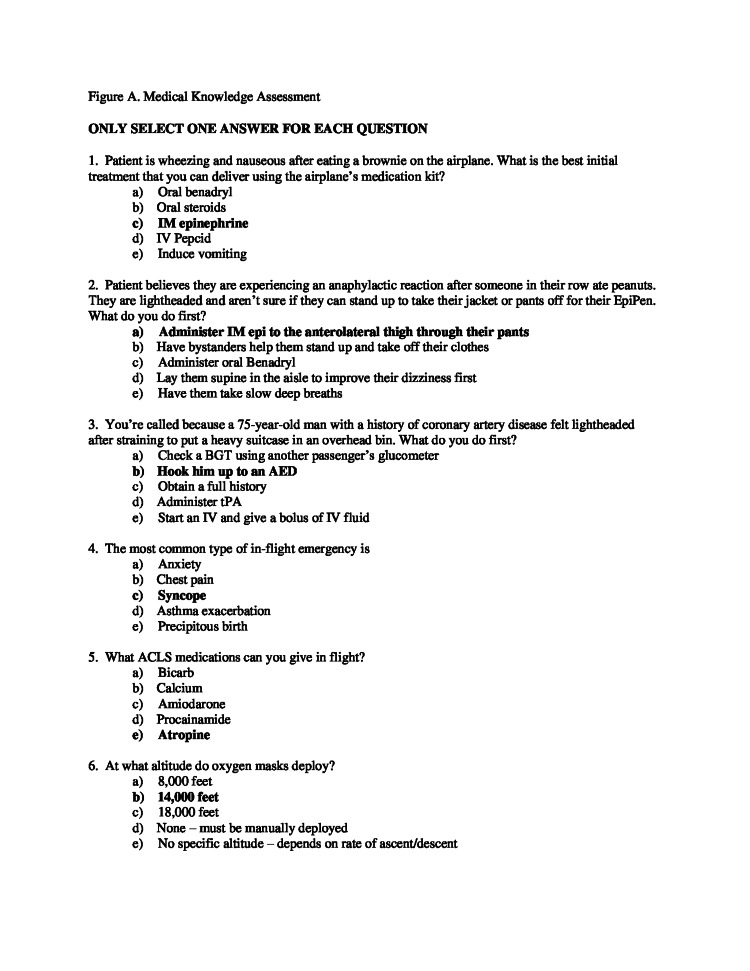
Medical knowledge test (part one) Abbreviations: IM=intramuscular, IV=intravenous, BGT=blood glucose test, AED=automated external defibrillator, tPA=tissue plasminogen activator, ACLS=advanced cardiac life support

**Figure 3:**
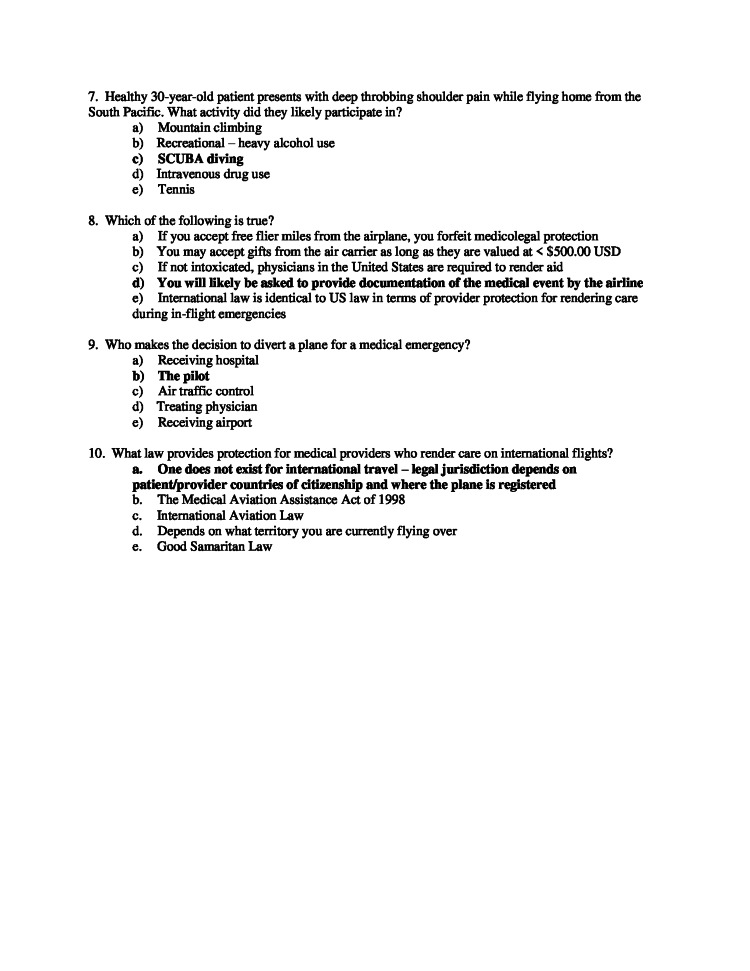
Medical knowledge test (part two) Abbreviations: SCUBA=self contained underwater breathing apparatus, USD=United States Dollar, US=United States
